# Does index tumor predominant location influence prognostic factors in radical prostatectomies?

**DOI:** 10.1590/S1677-5538.IBJU.2016.0335

**Published:** 2017

**Authors:** Athanase Billis, Leandro L. L. Freitas, Larissa B. E. Costa, Camila M. de Angelis, Kelson R. Carvalho, Luis A. Magna, Ubirajara Ferreira

**Affiliations:** 1 Departamento de Patologia, Faculdade de Ciências Médicas da Universidade Estadual de Campinas (Unicamp), Campinas, SP, Brasil;; 2 Departamento de Genética Médica/Bioestatística, Faculdade de Ciências Médicas da Universidade Estadual de Campinas (Unicamp), Campinas, SP, Brasil;; 3 Departamento Urologia, Faculdade de Ciências Médicas da Universidade Estadual de Campinas (Unicamp), Campinas, SP, Brasil

**Keywords:** Neoplasms, Prostate, Prostatectomy, Prostate-Specific Antigen

## Abstract

**Purpose:**

To find any influence on prognostic factors of index tumor according to predominant location.

**Materials and Methods:**

Prostate surgical specimens from 499 patients submitted to radical retropubic prostatectomy were step-sectioned. Each transverse section was subdivided into 2 anterolateral and 2 posterolateral quadrants. Tumor extent was evaluated by a semi-quantitative point-count method. The index tumor (dominant nodule) was recorded as the maximal number of positive points of the most extensive tumor area from the quadrants and the predominant location was considered anterior (anterolateral quadrants), posterior (posterolateral quadrants), basal (quadrants in upper half of the prostate), apical (quadrants in lower half of the prostate), left (left quadrants) or right (right quadrants). Time to biochemical recurrence was analyzed by Kaplan-Meier product-limit analysis and prediction of shorter time to biochemical recurrence using univariate and multivariate Cox proportional hazards model.

**Results:**

Index tumors with predominant posterior location were significantly associated with higher total tumor extent, needle and radical prostatectomy Gleason score, positive lymph nodes and preoperative prostate-specific antigen. Index tumors with predominant basal location were significantly associated with higher preoperative prostate-specific antigen, pathological stage higher than pT2, extra-prostatic extension, and seminal vesicle invasion. Index tumors with predominant basal location were significantly associated with time to biochemical recurrence in Kaplan-Meier estimates and significantly predicted shorter time to biochemical recurrence on univariate analysis but not on multivariate analysis.

**Conclusions:**

The study suggests that index tumor predominant location is associated with prognosis in radical prostatectomies, however, in multivariate analysis do not offer advantage over other well-established prognostic factors.

## INTRODUCTION

In a previous study we showed that total and index tumor extent were significantly associated with higher preoperative prostate specific antigen (PSA), clinical stage T2, pathological stage greater than T2, positive surgical margin (PSM) and higher radical prostatectomy (RP) Gleason score (1).Total and index tumor extent predicted time to biochemical recurrence (TBCR) following RP on univariate analysis. However, only dominant nodule (index tumor) extent was an independent predictor of TBCR on multivariate analysis. The study suggested that any type of tumor extent estimate in surgical specimens should be related to the dominant nodule (index tumor) and not to total tumor extent.

The aim of this study is to find any influence on prognostic factors related to location of index tumor.

## MATERIALS AND METHODS

This retrospective study was based on 499 consecutive patients submitted to radical retropubic prostatectomy by one surgeon (UF). Several clinicopathological variables were studied.

After RP, serum PSA from all patients was drawn every 3 months during the first year, every 6 months during the second year, and annually thereafter. No patient of this series had radiotherapy or androgen manipulation before or after surgery until biochemical recurrence (BCR) was observed. Total serum PSA was measured utilizing previous validated Immulite® PSA kit. BCR following surgery was considered as PSA ≥0.2ng/mL with a second confirmatory level of PSA >0.2ng/mL according to recommendation of the American Urological Association (2). Patients without evidence of BCR were censored at last follow-up. The present study was approved by the Institutional Committee of Ethics of our Institution.

The surgical specimens were step-sectioned at 3 to 5mm intervals and totally embedded in paraffin. A mean of 32 paraffin blocks were processed and 6µm sections from each block were stained with hematoxylin and eosin. Each transverse section of the prostate was subdivided into 2 anterolateral and 2 posterolateral quadrants. Using the cone method, 8 sections from the bladder neck and 8 sections from the apex were obtained.

Gleason grading was considered from the overall tumor of the surgical specimen. PSM was defined as cancer cells in contact with the inked specimen surface. Extra-prostatic extension (EPE) was diagnosed whenever cancer was seen in adipose tissue and, in case of desmoplastic response, whenever a protuberance corresponding to extension of tumor into peri-prostatic tissue was seen. Seminal vesicle (SV) invasion occurred whenever there was involvement of the muscular coat. Tumor extent at RP was evaluated by a semi-quantitative point-count method previously described (3). Briefly, drawn on a sheet of paper, each quadrant of the transverse sections contained 8 equidistant points. During the microscopic examination of the slides, the tumor area was drawn on the correspondent quadrant seen on the paper. At the end of the examination the amount of positive points represented an estimate of the tumor extent. Total tumor extent was recorded as the total sum of positive points from all transverse quadrants. Index tumor extent (dominant nodule) was recorded as the maximum number of positive points from the most extensive area of cancer present in the quadrants.

From a total of 499 patients, index tumor was considered as predominantly anterior (located in anterolateral quadrants) in 110 prostates, posterior (located in posterolateral quadrants) in 235 prostates, basal (located in quadrants of the upper half of the prostate) in 117 prostates, apical (located in quadrants of the lower half of the prostate) in 279 prostates, left side of the prostate (located in left quadrants) in 155 prostates, and right side of the prostate (located in right quadrants) in 180 prostates. Index tumor was defined as the most extensive tumor area (largest nodule) in the surgical specimen. Total number of patients in each location group is not the same. The reason, for example, is that predominant right side index tumors may be located predominantly in different locations: basal or apical, and anterior or posterior. Extensive tumors equally distributed between the studied locations were excluded for analysis.

The clinicopathologic findings included: age, clinical staging (T1c, and T2), pathological staging (pT2, and pT3a/pT3b), preoperative PSA, prostate weight, PSA density, nodular hyperplasia, total tumor extent, needle Gleason score, RP Gleason score, PSM, EPE, SV invasion, and positive lymph nodes.

**Figure 1:**
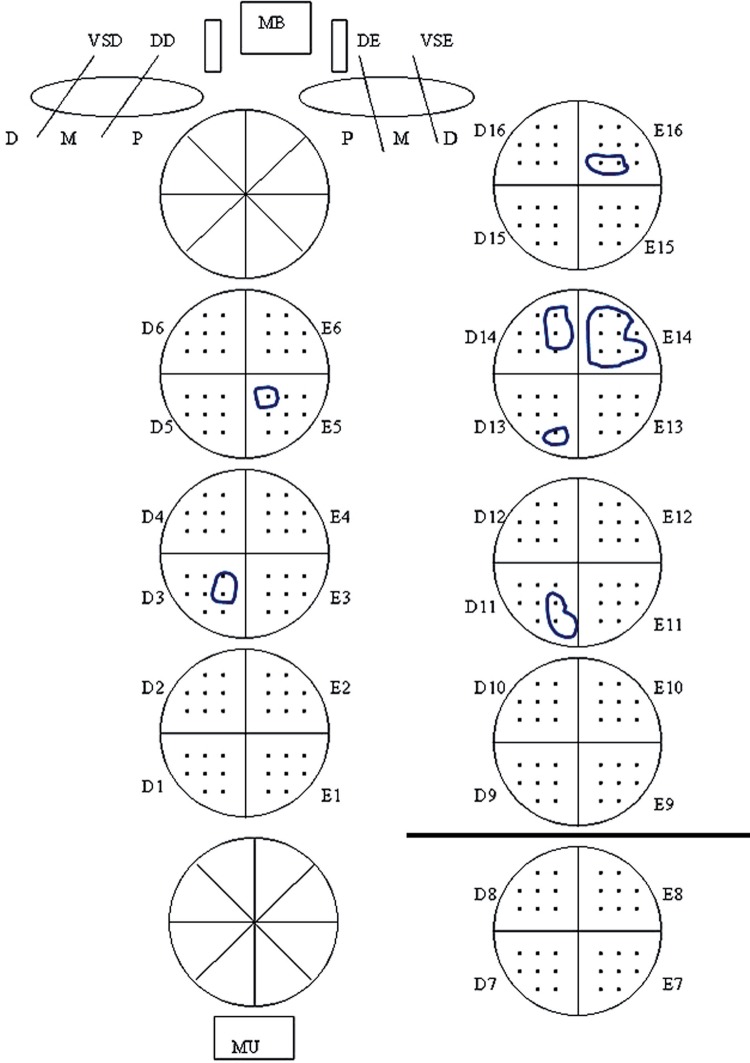
Semiquantitative point-count method to evaluate tumor extent. In this case total tumor extent was recorded as 17 positive points. Quadrant E14 shows largest single cancer focus or dominant nodule of all quadrants, recorded as 7 index tumor positive points. The tumor is predominantly basal (located in one quadrant of the upper half of the prostate). The horizontal line divides the prostate in quadrants located in upper and lower half of the prostate.

Figure-1 shows the drawing included in the pathology report with 8 equidistant points per quadrant. Total tumor extent was recorded as the total sum of the positive points of all transverse quadrants. Index tumor extent (dominant nodule) was recorded as the maximum number of positive points for the largest single focus of cancer in the quadrants. In this particular example, index tumor was in quadrant E14 and located predominantly at the base (upper half of the prostate).

### Statistical analysis

The data were analyzed using the Chi-square and the Fisher exact test for comparison of proportions, the Mann-Whitney test for comparison of means, and the Kaplan-Meier product-limit analysis for the TBCR using the log-rank test for comparison between the groups. A univariate and multivariate Cox stepwise logistic regression model was used to identify significant predictors of shorter TBCR. The relative importance of the prognostic variables was measured by the Wald test. The P-values were two-sided at the significance level of <0.05. All statistical analyses were performed using the commercial available PASW Statistics (SPSS) 18.0.

## RESULTS

### Clinicopathological Findings

Index tumors with predominant posterior location were significantly associated with higher total tumor extent, needle and RP Gleason score, positive lymph nodes and preoperative PSA (the latter in the limit of significance) (Table-1).

**Table 1:** Clinicopathological features of 345 patients by index tumor predominant location.

Feature	Anterior (n=110)	Posterior (n=235)	p Value
Mean ± SD age/median (range)	63.63 ± 6.45/65 (45-75)	62.89 ± 6.73/64 (43-76)	0.290 (Mann-Whitney test)
**No. race (%)**			
Whites	86 (78.2)	188 (81%)	0.563 (Fisher exact test)
African-Brazilians	24 (21.8)	44 (19%)	
**No. clinical stage (%)**			
T1c	65 (60.7)	129 (55.8)	0.410 (Fisher exact test)
T2	42 (39.3)	102 (44.2)	
Mean ± SD pre-op PSA/median (range)	8.03 ± 4.61/7.04 (0.6-22)	9.42 ± 5.64/8 (1.22-35)	0.050 (Mann-Whitney test)
Mean ± SD prostate weight/median (range)	39.18 ± 21/35 (10-130)	40.42 ± 21.81/35 (15-190)	0.524 (Mann-Whiteny test)
Mean ± SD PSA density/median (range)	0.24 ± 0.17/0.19 (0.02-.87)	0.35 ± 1.26/0.22 (0.04-19.25)	0.119 (Mann-Whitney test)
**No. nodular hyperplasia (%)**			
Neg	33 (30)	53 (22.9)	0.183 (Fisher exact test)
Pos	77 (70)	178 (77.1)	
Mean ± SD tumor extent/median (range)	22.97 ± 19.62/19 (1-94)	29.26 ± 25.91/23 (1-147)	0.040 (Mann-Whitney test)
Mean ± SD needle Gleason score/median (range)	6.30 ± 0.64/6 (4-9)	6.51 ± 0.68/6 (6-9)	0.007 (Mann-Whitney test)
Mean ± SD RP Gleason score/median (range)	6.53 ± 0.57/7 (5-8)	6.82 ± 0.74/7 (4-9)	<0.001 (Mann-Whitney test)
**No. surgical margin at any location (%)**			
Neg	66 (60)	120 (51.3)	0.134 (Fisher exact test)
Pos	44 (40)	114 (48.7)	
**No. Extra-prostatic extension (%)**			
Neg	85 (77.3)	174 (74)	0.594 (Fisher exact test)
Pos	25 (22.7)	61 (26)	
**No. seminal vesicle invasion (%)**			
Neg	105 (96.3)	215 (93.1)	0.325 (Fisher exact test)
Pos	4 (3.7)	16 (6.9)	
**No. pathological stage (%)**			
pT2	85 (77.3)	172 (73.2)	0.508 (Fisher exact test)
pT3a/pT3b	25 (22.7)	63 (26.8)	
**No. lymph nodes (%)**			
Not resected	64 (58.2)	107 (45.5)	0.040 (Chi-square test)
Neg	46 (41.8)	123 (52.3)	
Pos	0 (0)	5 (2.2)	

Index tumors with predominant basal location were significantly associated with higher preoperative PSA, pathological stage higher than pT2, EPE, and SV invasion (Table-2).

**Table 2:** Clinicopathological features of 396 patients by index tumor predominant location.

Feature	Basal (n=117)	Apical (n=279)	p Value
Mean ± SD age/median (range)	62.98 ± 6.40/64 (45-75)	62.96 ± 6.49/64 (42-76)	0.974 (Mann-Whitney test)
**No. race (%)**			
Whites	94 (81.0)	223 (80.5)	>0.999 (Fisher exact test)
African-Brazilians	22 (19.0)	54 (19.5)	
**No. clinical stage (%)**			
T1c	58 (50.9)	151 (54.7)	0.505 (Fisher exact test)
T2	56 (49.1)	125 (45.3)	
Mean ±SD pre-op PSA/median (range)	10.73±7.41/8.6 (0.60-51)	9.08±5.49/7.76 (0.28-33)	0.047 (Mann-Whitney test)
Mean ± SD prostate weight/median (range)	40.94 ± 22.67/35 (11-130)	40.24 ± 28.44/35 (10-190)	0.985 (Mann-Whitney test)
Mean ± SD PSA density/median (range)	0.30 ± 0.24/0.24 (0.03-1.38)	0.33 ± 1.16/0.21 (0.01-19.25)	0.133 Mann-Whitney test)
**No. nodular hyperplasia (%)**			
Neg	38 (32.8)	71 (26)	0.177 (Fisher exact test)
Pos	78 (67.2)	202 (74)	
Mean ± SD tumor extent/median (range)	35.12 ± 35.66/24.50 (1-225)	31.24 ± 27.18/26 (1-158)	0.775 (Mann-Whitney test)
Mean ± SD needle Gleason score/median(range)	6.49 ± 0.77/6 (4-9)	6.49 ± 0.68/6 (5-9)	0.770 (Mann-Whitney test)
Mean ± SD RP Gleason score/median (range)	6.83 ± 0.87/7 (5-9)	6.76 ± 0.75/7 (5-9)	0.899 (Mann-Whitney test)
**No. surgical margin at bladder neck (%)**			
Neg	102 (90.3)	271 (97.8)	0.002 (Fisher exact test)
Pos	11 (9.7)	6 (2.2)	
**No. surgical margin at** **apex (%)**			
Neg	107 (94.7)	237 (85.3)	0.009 (Fisher exact test)
Pos	6 (5.3)	41 (14.7)	
**No. extra-prostatic** **extension (%)**			
Neg	76 (65)	217 (77.8)	0.012 (Fisher exact test)
Pos	41 (35)	12 (22.2)	
**No. seminal vesicle** **invasion (%)**			
Neg	96 (84.2)	265 (95.7)	<0.001 (Fisher exact test)
Pos	18 (15.8)	12 (4.3)	
**No. pathological stage (%)**			
pT2	76 (65)	215 (77.1)	0.017 (Fisher exact test)
pT3a/pT3b	41 (35)	64 (22.9)	
**No**. **lymph nodes (%)**			
Not resected	54 (46.2)	144 (51.6)	0.364 (Chi-square test)
Neg	58 (49.6)	129 (46.2)	
Pos	5 (4.3)	6 (2.2)	

Index tumors predominantly at right side were significantly associated with higher preoperative PSA and prostate weight (Table-3).

**Table 3:** Clinicopathological features of 335 patients by index tumor predominant location.

Feature	Left (n=155)	Right (n=180)	p Value
Mean ± SD age/median (range)	63.17 ± 6.83/64 (42-76)	63.02 ± 6.32/64 (46-76)	0.685 (Mann-Whitney test)
**No. race (%)**			
Whites	122 (78.7)	146 (82.0)	0.489 (Fisher exact test)
African-Brazilians	33 (21.3)	32 (18.0)	
**No. clinical stage (%)**			
T1c	75 (48.7)	104 (58.4)	0.079 (Fisher exact test)
T2	79 (51.3)	74 (41.6)	
Mean ± SD pre-op PSA/median (range)	8.44 ± 5.16/7.2 (0.28-35)	9.80 ± 5.99/8 (0.6-41)	0.028 (Mann-Whitney test)
Mean ± SD prostate weight/median (range)	35.86 ± 18.87/30 (10-190)	40.96 ± 22.5/35 (11-185)	0.017 (Mann-Whitney test)
Mean ± SD PSA density/median (range)	0.27 ± 0.19/0.22 (0.01-1.17)	0.28 ± 0.22/0.22 (0.03-1.38)	0.589 (Mann-Whitney test)
**No. nodular hyperplasia**			
Neg	47 (30.9)	45 (25.1)	0.269 (Fisher exact test)
Pos	105 (69.1)	134 (74.9)	
Mean ± SD tumor extent/median (range)	29.17 ± 26.03/22 (1-127)	28.88 ± 26.58/24 (1-151)	0.866 (Mann-Whitney test)
Mean ± SD needle Gleason score/median (range)	6.49 ± 0.73/6 (5-9)	6.53 ± 0.69/6 (6-9)	0.435 (Mann-Whitney test)
Mean±SD RP Gleason score/median (range)	6.76 ± 0.69/7 (5-9)	6.78 ± 0.71/7 (4-9)	0.676 (Mann-Whitney test)
**No. surgical margin at any location (%)**			
Neg	91 (59.1)	91 (50.6)	0.124 (Fisher exact test)
Pos	63 (40.9)	89 (49.4)	
**No. Extra-prostatic extension (%)**			
Neg	110 (71)	129 (71.7)	0.904 (Fisher exact test)
Pos	45 (29)	51 (28.3)	
**No. seminal vesicle invasion (%)**			
Neg	143 (94.1)	165 (92.2)	0.525 (Fisher exact test)
Pos	9 (5.9)	14 (7.8)	
**No. pathological stage (%)**			
pT2	110 (71)	127 (70.6)	>0.999 (Fisher exact test)
pT3a/pT3b	45 (29)	53 (29.4)	
**No. lymph nodes (%)**			
Not resected	63 (40.6)	91 (50.6)	0.192 (Chi-square test)
Neg	88 (56.8)	85 (47.2)	
Pos	4 (2.6)	4 (2.2)	

### Time to biochemical recurrence

Index tumor with predominant anterior vs. posterior location

From a total of 345 patients following RP, 102 (29.6%) patients had BCR at a mean, median and range follow-up of 28, 15, and 1-158 months; 226 (65.5%) censored men remained at risk at a mean, median and range follow-up of 54, 44, and 1-169 months, respectively; and, 17 (4.9%) men had no serum PSA data.

**Figure 2:**
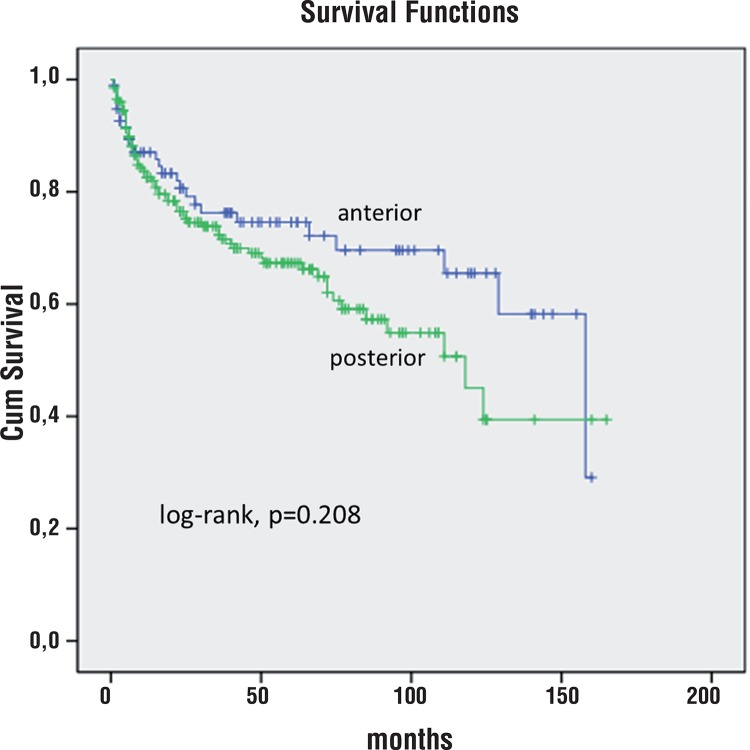
Kaplan-Meier product limit analysis shows time to PSA biochemical progression-free outcome by index tumor anterior vs posterior predominant location. Cum, cumulative.

At 5 years of follow-up, 74% of patients with predominantly anterior index tumor were free of BCR vs. 67% of patients with predominantly posterior index tumor (log-rank, p=0.208, Figure-2).

### Index tumor with predominant basal vs. apical location

From a total of 396 patients following RP, 125 (31.6%) patients had BCR at a mean, median and range follow-up of 25, 10, and 1-158 months; 256 (64.6%) censored men remained at risk at a mean, median and range follow-up of 54, 43, and 1-169 months, respectively; and, 15 (3.8 %) men had no serum PSA data.

**Figure 3:**
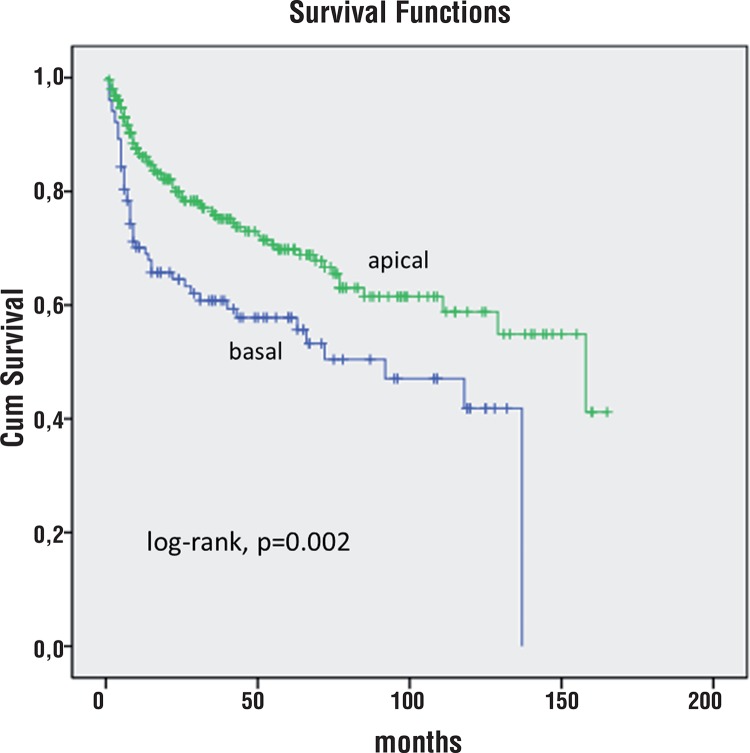
Kaplan-Meier product limit analysis shows time to PSA biochemical progression-free outcome by index tumor basal vs apical predominant location. Cum, cumulative.

At 5 years of follow-up, 59% of patients with predominantly basal index tumor were free of BCR vs. 70% of patients with predominantly apical index tumor (log-rank, p=0.002, Figure-3).

### Index tumor with predominant left vs. right location

From a total of 335 patients following RP, 103 (30.7 %) patients had BCR at a mean, median and range follow-up of 25, 13, and 1-129 months; 218 (65.1%) censored men remained at risk at a mean, median and range follow-up of 54, 43, and 1-169 months, respectively; and, 14 (4.2%) men had no serum PSA data.

**Figure 4:**
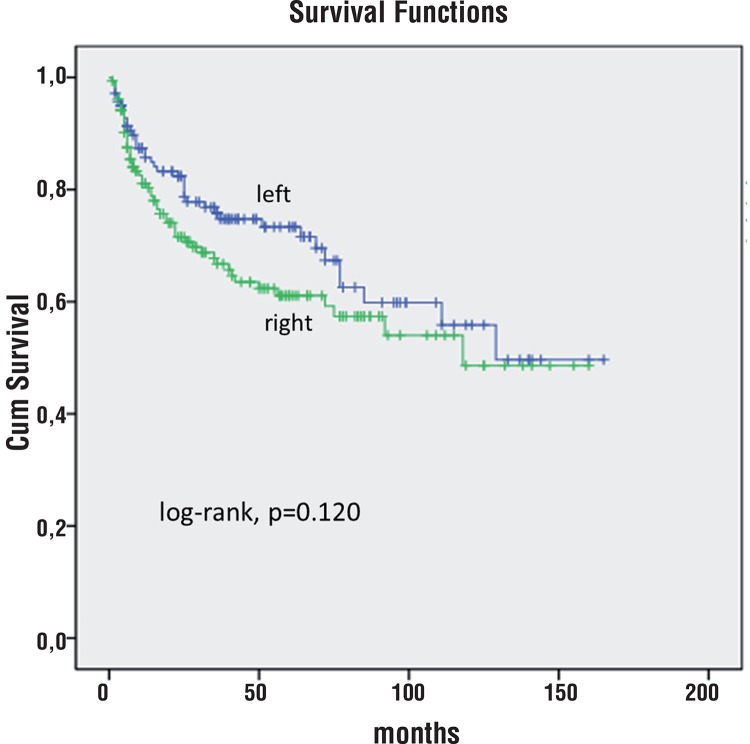
Kaplan-Meier product limit analysis shows time to PSA biochemical progression-free outcome by index tumor left vs right predominant location. Cum, cumulative.

At 5 years of follow-up, 79% of patients with predominantly left index tumor were free of BCR vs. 61% of patients with predominantly right index tumor (log-rank, p=0.120, Figure-4).

### Risk of shorter time to biochemical recurrence

In univariate Cox regression analysis (Table-4), PSA density, needle Gleason score, preoperative PSA, predominant index tumor basal location, EPE, total tumor extent, pathological stage greater than T2, RP Gleason score, SV invasion, and PSM were significantly predictive of shorter TBCR.

**Table 4:** Cox univariate and multivariate proportional hazard analysis of several clinicopathological factors predicting shorter time to biochemical recurrence after radical prostatectomy.

Predictors	HR (95% CI)	Wald test	p Value
	Univariate		
			
Age	0.997 (0.974-1.021)	0.063	0.802
Race	0.835 (0.551-1.264)	0.726	0.394
Clinical stage	1.174 (0.860-1.603)	1.021	0.312
Nodular hyperplasia	0.828 (0.594-1.154)	1.237	0.266
Index tumor: ant vs post	1.316 (0.855-2.025)	1.596	0.212
Index tumor: left vs right	1.361 (0.920-2.015)	2.377	0.123
Positive lymph nodes	2.002 (0.865-4.633)	2.631	0.105
Prostate weight	1.006 (1.000-1.013)	3.415	0.065
PSA density	1.812 (1.048-3.133)	4.530	0.033
Needle Gleason score	1.337 (1.077-1.659)	6.951	0.008
Pre-op PSA	1.026 (1.008-1.043)	8.605	0.003
Index tumor: basal vs apical	1.745 (1.218-2.500)	9.214	0.002
Extra-prostatic extension	1.708 (1.239-2.356)	10.674	0.001
Tumor extent	1.006 (1.003-1.010)	10.953	0.001
Pathological stage >T2	1.771 (1.287-2.438)	12.311	<0.001
RP Gleason score	1.422 (1.169-1.728)	12.471	<0.001
Seminal vesicle invasion	2.781 (1.832-4.223)	23.035	<0.001
Positive surgical margin	2.366 (1.709-3.275)	26.902	<0.001
			
	Multivariate		
			
Tumor extent	0.999 (0.992-1.006)	0.085	0.771
RP Gleason score	1.057 (0.784-1.426)	0.132	0.717
PSA density	0.802 (0.345-1.863)	0.263	0.608
Pathological stage >T2	0.438 (0.048-4.010)	0.533	0.465
Extra-prostatic extension	0.340 (0.770-0.451)	0.912	0.340
Pre-op PSA	1.029 (0.982-1.069)	1.268	0.260
Index tumor: basal vs apical	0.764 (0.512-1.139)	1.751	0.186
Needle Gleason score	1.293 (0.982-1703)	3.348	0.067
Seminal vesicle invasion	2.326 (1.314-4.120)	8.384	0.004
Positive surgical margin	2.150 (1.455-3.177)	14.761	<0.001

In multivariate analysis (Table-4) including all significant predictors in univariate analysis, only SV invasion and PSM were independent predictors of shorter TBCR. In all models we used the backward stepwise logistic regression method.

## DISCUSSION

Index tumors with predominant posterior location (posterolateral quadrants) comprise most part of the peripheral zone (PZ), and with predominant anterior location (anterolateral quadrants) most part of the transition zone (TZ). Index tumors with predominant posterior location were significantly associated with higher total tumor extent, needle and RP Gleason score, positive lymph nodes and preoperative serum PSA (the latter in the limit of significance).

Index tumors with predominant basal location were significantly associated with higher preoperative serum PSA, pathological stage higher than pT2, EPE, SV invasion, TBCR in Kaplan-Meier estimates and significantly predicted shorter TBCR on univariate analysis but not on multivariate analysis. There are several studies comparing index tumor in PZ location with index tumor with TZ location but to the best of our knowledge we did not find any mention to basal or apical location.

The 2009 ISUP (International Society of Urological Pathology) meeting failed to a consensus on the dominant pathological parameters of tumor extension or volume, Gleason score, or staging that define index tumor (4). However, most of the participants considered to be the largest nodule in multifocal disease. Moreover, in most of the cases, it corresponds also to the highest Gleason score in accordance with the global Gleason score.

Prostate cancer emerges as an evolutionary process often leading to multiple competing subclones within a single primary index tumor. This evolutionary process culminates in the formation of metastases. However, although the hypothesis that each metastasis originates from a single tumor cell is generally supported, several studies have supported the existence of polyclonal seeding from an interclonal cooperation between multiple subclones. These latter findings bring insights to find the “true” index lesion by looking on genetic, epigenetic and proteomic alterations (5, 6).

In Al-Ahmadie et al. (7) study in radical prostatectomies, 35.5% cancers were considered as originating from the TZ. This percentage is very similar to ours (31.9%). TZ tumors seem to be of lower degree of biologic aggressiveness (8). In radical prostatectomies, Grignon et al. (9) found that the mean Gleason score for the PZ and TZ tumors was 6.7 and 5.6, respectively (p<0.001). Gleason score also was higher in PZ cancers in the study by Lee et al. (10). In our study, the mean Gleason score in index tumors posteriorly located vs. anteriorly located was significantly higher in needle biopsies (p=0.007) and in RP (p<0.001).

In Lee et al. (10) study, 48% cancers originating in the PZ showed EPE, and 22% of cancers originating in the TZ. In our study, EPE was present in 26% and 22.7% of cancers located predominantly at posterior and anterior location, respectively (p=0.594). However, EPE was present in 35% and 22.2% of cancers located predominantly at basal and apical location, respectively (p=0.012). Basal tumor location was significantly associated with higher serum PSA (0.047) as well as index tumors with posterior location (the latter in the limit of significance, p=0.050). Interestingly, predominantly right side index tumors had significantly higher serum PSA (p=0.028) as well as higher prostate weight (p=0.017).

Greene et al. (8) found that SV invasion arose from 19% of the PZ but none of the TZ cancers. In our study, there was no significant difference in SV invasion comparing predominantly anterior with posterior located tumors (p=0.325). A very significant difference was found comparing basal with apical location. SV invasion was present in 15.8% of tumors located at the base and in 4.3% of tumors located at the apex (p<0.001).

In Noguchi et al. (11) study, Kaplan-Meier curves showed that at 5 years of follow-up 49.2% of men with PZ cancer had undetectable PSA compared with 71.5% of those with TZ cancer (log rank, p=0.0002). Stamey et al. (12) reported a 5-year disease-free survival rate of 53% in men with PZ and 81% in those with TZ cancers. Sakai et al. (13) showed that there was no significant difference in biochemical recurrence-free survival between patients with TZ and PZ cancers. Augustin et al. (14) found that the location of prostate cancer in the TZ was associated with a greater overall biochemical cure rate after RP. However, they found that it was not an independent prognostic factor on multivariate analysis. Therefore, the authors concluded that knowledge about zonal location of prostate cancer offers no advantage over the well-established prognostic factors in predicting disease recurrence. Chun et al. (15) showed that in multivariate Cox models, the rate of BCR was not significantly different between TZ and PZ prostate cancers (p=0.4).

In our study, the Kaplan-Meier curves did not show any significant difference comparing anterior vs posterior index tumor location. At 5 years of follow-up, 74% of patients with predominantly anterior index tumor were free of BCR vs 67% of patients with predominantly posterior index tumor (log-rank, p=0.208, Figure-2). On the other hand, at 5 years of follow-up, 59% of patients with predominantly basal index tumor were free of BCR vs 70% of patients with predominantly apical index tumor (log-rank, p=0.002 Figure-3). In univariate analysis, predominantly basal tumor location had significantly shorter TBCR (p=0.002) but not in multivariate analysis (p=0.186). Only needle SV invasion (pT3b), and PSM were independent predictors of shorter TBCR.

Iremashvili et al. (16) found that the rates of PSM were similar in men with TZ and mixed tumors and were significantly higher than those with PZ tumors. In index tumors located at the TZ, Van de Voorde et al. (17) found that EPE, SV involvement, PSMs, and lymph node metastasis were seen in the TZ cancer group in 33%, 17%, 29%, and 4%, respectively versus 58%, 20%, 48%, and 6% in the PZ cancer group. In our cohort of patients who had lymph nodes resected, metastasis occurred in 2.2% of posteriorly located tumors and 0% anteriorly; 4.3% in basal tumors and 2.2% in apical located tumors.

Comparing anteriorly and posteriorly located tumors, Mygatt et al. (18) found that there was no difference between mean age, body mass index, racial distribution, family history, number of previous biopsies, clinical Gleason sum or pathological stage in the two groups. Lallas et al. (19) showed that patients with PSM were subsequently found to have higher risk of biochemical recurrence. O’Neil et al. (20) comparing TZ tumors with PZ tumors found that the formers were larger, more frequently lower grade, organ confined, and preferentially involved the bladder neck (49% vs 6%, p<0.001). Tumor zonality was not associated with BCR for the entire cohort. PSA recurrence in patients with histologically confirmed PSMs after RP was independent of the zonal location of the index tumor.

We did not find any racial difference considering all locations studied. Anterior vs posterior, and left vs right location did not show any statistical significant difference associated with PSM. However, in predominant basal location vs apical location the frequency of bladder neck PSM was 9.7% and 2.2% (p=0.002), respectively; and, apical PSM was 5.3% and 14.7% (p=0.009), respectively.

Predominant basal tumor location was significantly associated with higher pathologic stage. EPE was present in 35% of basal tumors vs 22.2% apical tumors (p=0.012), and SV invasion in 15.8% vs 4.3%, respectively (p<0.001). The finding of SV invasion in a RP specimen markedly diminishes the likelihood of cure. Possible routes of SV invasion are: 1) extension into soft tissue adjacent to the SV and then into the SV; 2) invasion via the sheath of the ejaculatory duct, penetrating the muscular wall of the ejaculatory duct, or extending up the ejaculatory duct wall into the SV muscle wall; 3) direct invasion of the SV; or 4) discontinuous metastases. There are conflicting studies as to whether the first or second method is most common (21-23). Metastases are the least common mode of spread.

Epstein et al. (23) reported the findings of 60 men who had undergone radical retropubic prostatectomy and whose tumors demonstrated isolated SV invasion. In their study the most frequent route of SV invasion (34/60 patients, 56.7%) was tumor extension out of the prostate at the base of the gland into the peri-seminal vesicle tissue, with subsequent invasion into the muscular wall of the SV. In favor of this finding is the fact that in unilateral invasion of the SV most frequently there is ipsilateral EPE and in bilateral invasion most frequently there is bilateral EPE (24). Besides the anatomic proximity, the finding in our study of a significant higher EPE in predominantly basal tumor location, favors that extension into soft tissue adjacent to the SV with subsequent invasion into the muscular wall is the most frequent route of SV invasion.

Some study limitations warrant discussion. Standard pathological evaluation of the index tumor may not be parallel to the axis and be a confounding location considering the tridimensional aspect of the lesion. Follow-up of the patients studied could be longer and the number of patients higher. If we had incorporated additional variables in the Cox model, such as tumor extent on biopsy, preoperative PSA velocity and others, results could have been different. Therefore, other studies are needed that incorporate these variables as well as studies that include basal and apical index tumor predominant location for the sake of comparison with our results.

## CONCLUSIONS

Index tumors with predominant posterior location were significantly associated with higher total tumor extent, needle and RP Gleason score, positive lymph nodes and preoperative PSA. Index tumors with predominant basal location were significantly associated with higher preoperative PSA, pathological stage higher than pT2, EPE, SV invasion, TBCR in Kaplan-Meier estimates and significantly predicted shorter TBCR on univariate analysis but not on multivariate analysis. The study suggests that index tumor predominant location is associated with prognosis in radical prostatectomies, however, in multivariate analysis do not offer advantage over other well-established prognostic factors.

## ABBREVIATIONS

PSA: Prostate specific antigen
RP: Radical prostatectomy
SD: Standard deviation
CI: Confidence interval
HR: Hazard
BCR: Biochemical recurrence
TBCR: Time to BCR
PSM: Positive surgical margin
EPE: Extra-prostatic extension
SV: Seminal vesicle
PZ: Peripheral zone
TZ: Transitional zone

## References

[1] Billis A, Meirelles LR, Freitas LL, Polidoro AS, Fernandes HA, Padilha MM (2013). Prostate total tumor extent versus index tumor extent--which is predictive of biochemical recurrence following radical prostatectomy?. J Urol.

[2] Cookson MS, Aus G, Burnett AL, Canby-Hagino ED, D’Amico AV, Dmochowski RR (2007). Variation in the definition of biochemical recurrence in patients treated for localized prostate cancer: the American Urological Association Prostate Guidelines for Localized Prostate Cancer Update Panel report and recommendations for a standard in the reporting of surgical outcomes. J Urol.

[3] Billis A, Magna LA, Ferreira U (2003). Correlation between tumor extent in radical prostatectomies and preoperative PSA, histological grade, surgical margins, and extraprostatic extension: application of a new practical method for tumor extent evaluation. Int Braz J Urol.

[4] Kwast TH van der, Amin MB, Billis A, Epstein JI, Griffiths D, Humphrey PA (2011). International Society of Urological Pathology (ISUP) Consensus Conference on Handling and Staging of Radical Prostatectomy Specimens. Working group 2: T2 substaging and prostate cancer volume. Mod Pathol.

[5] Gundem G, Van Loo P, Kremeyer B, Alexandrov LB, Tubio JM, Papaemmanuil E (2015). The evolutionary history of lethal metastatic prostate cancer. Nature.

[6] Van Etten JL, Dehm SM (2016). Clonal origin and spread of metastatic prostate cancer. Endocr Relat Cancer.

[7] Al-Ahmadie HA, Tickoo SK, Olgac S, Gopalan A, Scardino PT, Reuter VE (2008). Anterior-predominant prostatic tumors: zone of origin and pathologic outcomes at radical prostatectomy. Am J Surg Pathol.

[8] Greene DR, Wheeler TM, Egawa S, Dunn JK, Scardino PT (1991). A comparison of the morphological features of cancer arising in the transition zone and in the peripheral zone of the prostate. J Urol.

[9] Grignon DJ, Sakr WA (1994). Zonal origin of prostatic adenocarcinoma: are there biologic differences between transition zone and peripheral zone adenocarcinomas of the prostate gland?. J Cell Biochem Suppl.

[10] Lee F, Siders DB, Torp-Pedersen ST, Kirscht JL, McHugh TA, Mitchell AE (1991). Prostate cancer: transrectal ultrasound and pathology comparison. A preliminar study of outer gland (peripheral and central zones) and inner gland (transition zone) cancer. Cancer.

[11] Noguchi M, Stamey TA, Neal JE, Yemoto CE (2000). An analysis of 148 consecutive transition zone cancers: clinical and histological characteristics. J Urol.

[12] Stamey TA, Sözen TS, Yemoto CM, McNeal JE (1998). Classification of localized untreated prostate cancer based on 791 men treated only with radical prostatectomy: common ground for therapeutic trials and TNM subgroups. J Urol.

[13] Sakai I, Harada K, Kurahashi T, Yamanaka K, Hara I, Miyake H (2006). Analysis of differences in clinicopathological features between prostate cancers located in the transition and peripheral zones. Int J Urol.

[14] Augustin H, Hammerer PG, Blonski J, Graefen M, Palisaar J, Daghofer F (2003). Zonal location of prostate cancer: significance for disease-free survival after radical prostatectomy?. Urology.

[15] Chun FK, Briganti A, Jeldres C, Erbersdobler A, Schlomm T, Steuber T (2007). Zonal origin of localized prostate cancer does not affect the rate of biochemical recurrence after radical prostatectomy. Eur Urol.

[16] Iremashvili V, Pelaez L, Jordá M, Manoharan M, Rosenberg DL, Soloway MS (2012). Prostate cancers of different zonal origin: clinicopathological characteristics and biochemical outcome after radical prostatectomy. Urology.

[17] Van de Voorde WM, Van Poppel HP, Verbeken EK, Oyen RH, Baert LV, Lauweryns JM (1995). Morphologic and neuroendocrine features of adenocarcinoma arising in the transition zone and in the peripheral zone of the prostate. Mod Pathol.

[18] Mygatt J, Sesterhenn I, Rosner I, Chen Y, Cullen J, Morris-Gore T (2014). Anterior tumors of the prostate: clinicopathological features and outcomes. Prostate Cancer Prostatic Dis.

[19] Lallas CD, Fashola Y, Den RB, Gelpi-Hammerschmidt F, Calvaresi AE, McCue P (2014). Predictors of positive surgical margins after radical prostatectomy at a single institution: preoperative and pathologic factors, and the impact of surgeon variability and technique on incidence and location. Can J Urol.

[20] O’Neil LM, Walsh S, Cohen RJ, Lee S (2015). Prostate carcinoma with positive margins at radical prostatectomy: role of tumour zonal origin in biochemical recurrence. BJU Int.

[21] Villers AA, McNeal JE, Redwine EA, Freiha FS, Stamey TA (1990). Pathogenesis and biological significance of seminal vesicle invasion in prostatic adenocarcinoma. J Urol.

[22] Ohori M, Scardino PT, Lapin SL, Seale-Hawkins C, Link J, Wheeler TM (1993). The mechanisms and prognostic significance of seminal vesicle involvement by prostate cancer. Am J Surg Pathol.

[23] Epstein JI, Partin AW, Potter SR, Walsh PC (2000). Adenocarcinoma of the prostate invading the seminal vesicle: prognostic stratification based on pathologic parameters. Urology.

[24] Billis A, Teixeira DA, Stelini RF, Quintal MM, Guimarães MS, Ferreira U (2007). Seminal vesicle invasion in radical prostatectomies: which is the most common route of invasion?. Int Urol Nephrol.

